# N-end rule pathway inhibition assists colon tumor regression via necroptosis

**DOI:** 10.1038/mto.2016.20

**Published:** 2016-08-10

**Authors:** Pritha Agarwalla, Rajkumar Banerjee

**Affiliations:** 1 Biomaterials Group, CSIR-Indian Institute of Chemical Technology (CSIR-IICT), Hyderabad, India; 2 Academy of Scientific and Innovative Research (AcSIR), New Delhi, India

## Abstract

Recent study has shown that N-end rule pathway, an ubiquitin dependent proteolytic system, counteracts cell death by degrading many antisurvival protein fragments like BCL_xL_, BRCA1, RIPK1, etc. Inhibition of the N-end rule pathway can lead to metabolic stabilization of proapoptotic protein fragments like RIPK1, thereby sensitizing cells to programmed cell death. Receptor interacting serine-threonine protein kinase-1 (RIPK1) is one of the upstream regulators of programmed necrosis known as necroptosis. Necroptosis is particularly gaining attention of cancer biologists as it provides an alternate therapeutic modality to kill cancer cells, which often evolve multiple strategies to circumvent growth inhibition by apoptosis. Utilizing the over expression of biotin receptor in cancer cells, herein, we report that coadministration of synthetic hetero-bivalent N-end rule inhibitor RFC11 and anticancer drug shikonin solubilized in a stable biotin receptor-targeted liposome exhibited significant synergistic antitumor effect in both subcutaneous and orthotopic mouse colon tumor model through induction of necroptosis with distinctive upregulation of RIPK1. Besides developing a newly targeted formulation for necroptosis induction, this report is the first *in vivo* evidence demonstrating that potent inhibition of N-end rule pathway can enhance therapeutic efficacy of conventional chemotherapeutics.

## Introduction

Despite several approaches to combat cancer, chemotherapy continues to be the most popular mode of treatment in inhibiting tumor growth. Apoptosis is the most exploited mechanism in chemotherapy-induced cancer cell death. However, a major roadblock of traditional chemotherapy is resistance of cancer cells to apoptosis.^1,2^ Thus, use of chemotherapeutics that target other nonapoptotic pathway in inhibiting proliferation of cancer cells is an attractive alternative for otherwise apoptosis-resistant cancer cells. Recently, necroptosis, a form of programmed necrosis has garnered lot of attentions as one of such therapeutic alternatives. Different stimuli can activate necroptosis and all of them converge at the interaction of the RIP1 and RIP3 kinases under conditions in which caspase-8 is not active.^3–6^ Currently, shikonin, a naturally occurring naphthoquinone purified from *Lithospermum erythrorhixon*, a Chinese medicinal herb, has gained considerable attention for its ability to induce apoptosis or necroptosis depending upon cell types, time and concentration used.^7–10^ It is already known that shikonin induces necroptosis in osteosarcoma, leukemia, and glioma cell lines by sequential upregulation of RIP kinases (RIPK1, RIPK3).^11–13^ Interestingly, this RIPK1 like many other protein fragments is degraded by N-end rule pathway.^14^ N-end rule, an ubiquitin dependent protein degradation pathway relates the *in vivo* half-life of a protein to the identity of its N-terminal residue.^15,16^ The pathway is related to ubiquitination and subsequent degradation of multiple cell cycle and apoptosis-related proteins.^17^ Caspases, the cysteine proteases cleave various target proteins like BRCA1, BID, TRAF1, RIPK1 to give protein fragments that are short lived N-end rule substrates.^14^ By destroying these proapoptotic fragments (including RIPK1), N-end rule downregulates their proapoptotic activities and thereby inhibits signal transduction that leads to cell death. Thus, in this context, N-end rule is a repressor of apoptosis at least in part through its ability to destroy such proapoptotic fragments. Hence, metabolic stabilization of any such fragment by even a partial ablation of N-end rule pathway in cancer cells could sensitize cells to chemotherapeutics. We have recently developed non-peptide based hetero-bivalent N-end rule pathway inhibitor called RFC11 (Scheme 1) that shows the ability to restore *in vivo* stability of various N-end rule substrates leading to various physiological changes.^18^ Therefore, combination of RFC11, which by inhibiting N-end rule pathway can stabilize RIPK1 with shikonin (already known to elevate expression of RIPK1 in certain cells), is likely to increase drug sensitivity of cancer cells leaving them vulnerable to apoptosis or necroptosis. Herein, we have exploited N-end rule pathway to induce synergistic anticancer effect (via induction of necroptosis) both *in vitro* and for the first time, in *in vivo* set up. However poor bioavailability, nonspecific tissue distribution, and rapid clearance by reticulo endothelial system are the major roadblocks in systemic administration of such hydrophobic drugs like shikonin and RFC11. In order to overcome such limitations of pristine drugs, targeted drug delivery systems (DDS) continues to remain a promising approach to combat cancer.^19–21^

Biotin (vitamin B7, vitamin H) is an essential micronutrient for normal cellular functions and is required in excess by various cancer cells to sustain their rapid proliferation. Biotin receptor is often found to be over expressed in a number of cancer cell lines of ovarian, colorectal, etc. origins and has emerged as a promising molecular marker for targeted drug delivery.^22–24^ Several research groups have developed different biotinylated therapeutics like biotin drug conjugates, biotinylated polymeric carriers for use as drug delivery vehicles or theranostic agents.^25–29^ In this study, we have developed a circulation stable liposomal formulation containing a biotin-based amphiphilic lipid, BIO-C18 (Scheme 1) that can stably entrap both RFC11 and shikonin and selectively accumulate to tumors. Moreover, by using an orthotopic as well as a subcutaneous model of CT26 colon adenocarcinoma in BALB/c mice, we have shown that administration of shikonin and RFC11 coentrapped in liposome of BIO-C18 can indeed inhibit the growth of solid tumor presumably via induction of necroptosis. In context of cancer treatment, the present findings suggests a new role of N-end rule inhibitor RFC11, which when co-treated with shikonin in a targeted liposomal formulation can significantly enhance drug sensitivity selectively in cancer cells both *in vitro* and *in vivo* through induction of necroptosis.

## Results

### Characterization of liposomes

**Figure 1:**
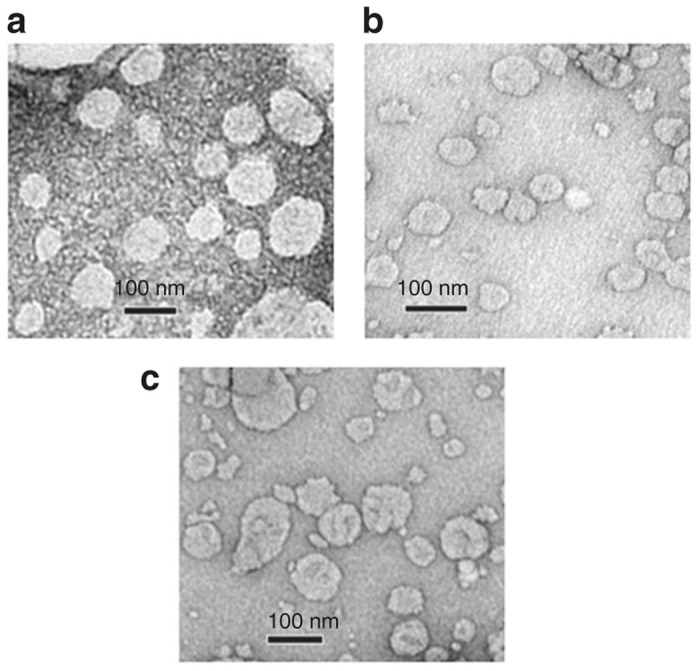
TEM images of liposomes of Bio-C18 lipid containing RFC11 (**a**), Shikonin (**b**), both RFC11 and shikonin (**c**).

Hydrodynamic diameters and zeta potentials of the biotinylated liposomal formulations containing only RFC11, only shikonin as well as both RFC11 and shikonin were measured by dynamic light scattering in sterilized water. The sizes of these formulations were found to be in the range of 150–220 nm and they exhibited a positive surface charge (22–35 mV) (Supplementary Table S1). The result of transmission electron microscopy (Figure 1) showed that liposomes were well-formed spherical vesicles and had compacted structure.

### Stability of liposomes in presence of serum

The serum stability profile of liposomes was measured by incubating the liposomes in 20 and 50% fetal bovine serum in phosphate buffer saline (PBS). Dynamic light scattering was used to measure changes in size of the liposome mixture at several time points. Supplementary Figure S5 shows size distribution of liposomes after incubation with serum was similar as separately measured and increased only 25% after 48 hours in 20% serum.

### Biotin receptor-mediated cellular uptake of liposomes

**Figure 2:**
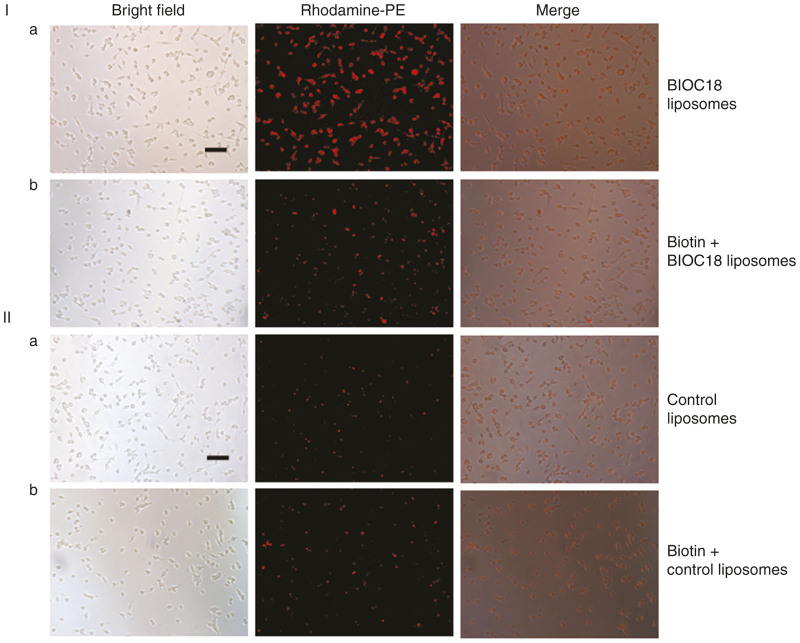
Microscopic images of CT26 cells. (**I**) Microscopic images showing cellular uptake of Rh-PE-labeled liposomes of BIO-C18 lipid (**a**) which decreased considerably upon pretreatment with 1 mmol/l biotin (**b**). (**II**) Cellular uptake of Rh-PE-labeled liposomes void of BIO-C18 (**a**) did not change when cells were preincubated with 1 mmol/l biotin (**b**). All the images were taken 2 hours after treatment with liposomes at 10× magnification (scale bar = 100 micron). The results shown are representative of three independent experiments.

Cellular uptake of Rh-PE-labeled liposomes of Bio-C18 lipid in CT26 cell line was observed by fluorescence microscopy. Interestingly, the cellular uptake of biotinylated liposome was found to be significantly higher than the nontargeting counterpart (Figure 2). This significantly higher cellular uptake of the liposomes of Bio-C18 lipid indicated receptor-mediated internalization. To confirm this, CT26 cells were presaturated with biotin (1 mmol/l) for 1 hour and it was observed that the uptake of liposomal formulation of Bio-C18 lipid was severely compromised but similar effect was not observed in case of liposomes without BIO-C18. Flow cytometric uptake data (Supplementary Figure S6) also revealed that cellular uptake of presently described liposome is severely compromised when CT26 cells were presaturated with biotin. To further consolidate our hypothesis that Rh-PE-labeled liposomes of Bio-C18 lipid presumably internalize through biotin receptor, we performed similar cellular uptake study in biotin receptor over expressed A549 and Hela cells as well as in basally expressing normal human foreskin fibroblast (HFF) cells. Cellular uptake of Rh-PE-labeled liposomes was compromised in A549 and Hela cells but not in HFF cells when cells were preincubated with biotin (Supplementary Figure S7). These observations support the notion that biotinylated liposome internalize into cells presumably via biotin receptor thus increasing the targeting efficiency of liposomes.

### *In vitro* cytotoxicity assay

**Figure 3:**
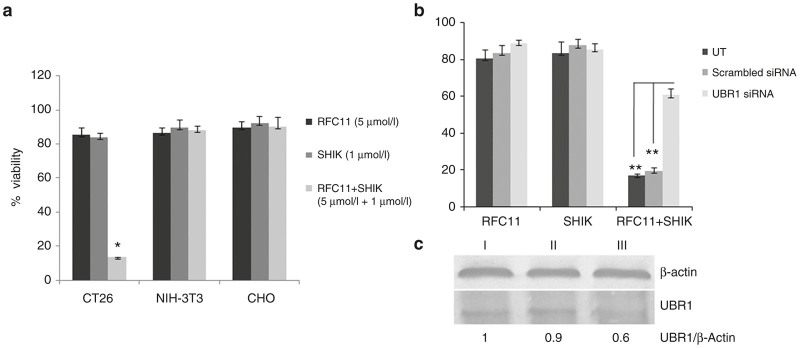
Cell viability studies: (**a**) N-end rule inhibitor RFC11 coencapsulated with shikonin in biotinylated liposomes exhibit synergistic cytotoxic effect in CT26 (mice colon adenocarcinoma) cells but not in non cancer cells (CHO, NIH-3T3). The asterisk (*) denotes *P* < 0.005 when compared with RFC11. (**b**) Cellular viability of siRNA untreated or siRNA (scrambled or UBR1) treated cells in presence of biotinylated liposomes containing RFC11 (5 μmol/l), shikonin (1 μmol/l), or both RF-C11 and shikonin (5+1 μmol/l). ** denotes *P* < 0.001. (**c**) UBR1 expression levels in CT26 cells, either untreated (I) or treated with scrambled siRNA (10 pmol/l) (II), or treated with UBR1 siRNA (10 pmol/l) (III).

We compared the toxicity of various liposomal formulation (containing RFC11 and/or shikonin) in cancerous (CT26, mice colon adenocarcinoma) as well as noncancerous (CHO, Chinese hamster ovary andNIH-3T3, mouse embryonic fibroblast) cells using standard MTT assay. Liposome of BIO-C18 lipid containing shikonin (1 μmol/l) or RFC11 (5 μmol/l) when treated individually imparted minimal cytotoxicity toward colon cancer cell but exhibited significant level of cytotoxicity when coencapsulated within the same liposome of Bio-C18 lipid (Figure 3a). Even cotreatment of RFC11 and shikonin individually entrapped in liposomes of Bio-C18 lipid exhibited similar synergistic cytotoxic effects in CT26 cells (Supplementary Figure S8). In contrast to cancer cells, noncancer cells did not show any significant cytotoxicity after 24 hours treatment of shikonin and RFC11 individually or in combination (Figure 3a). These data indicate that coadministration of RFC11 and shikonin in liposomal formulation of BIO-C18 synergistically induces cytotoxicity in cancer cell but not in noncancer cells. Also, as shown in Supplementary Figure S9, the CI value of 0.58 for combination of 5 µmol/l RFC11 and 1 µmol/l of shikonin, both entrapped in liposomal formulation, indicated very potent synergistic effect.

### Degradation of UBR1 desensitizes cancer cells and decreases synergistic cytotoxicity of shikonin and RFC11

UBR1 is one of the characterized E3 ligases involved in N-end rule pathway for ubiquitination of N-degrons. Through binding assays, we have previously established that RFC11 directly interacts with N-recognin UBR box of UBR1, which results in inhibition of N-degron’s ubiquitin-led degradation.^18^ To know the fate of cancer cells’ drug sensitivity after the depletion of UBR protein, we used specific siRNA against UBR1. Figure 3b shows that with the depletion of UBR1 proteins, RFC11 entrapped in biotinylated liposome could not enhance the therapeutic efficacy of shikonin. However, usual drug synergism was observed in siRNA-untreated and scrambled siRNA-treated cells. Clearly, UBR1’s presence is required for the RFC11-assisted enhanced cytotoxicity in cancer cells. The depletion of UBR1 protein by siRNA was confirmed by western blot analysis as shown in Figure 3c.

### Assessment of cellular cytotoxicity by flow cytometric study

**Figure 4:**
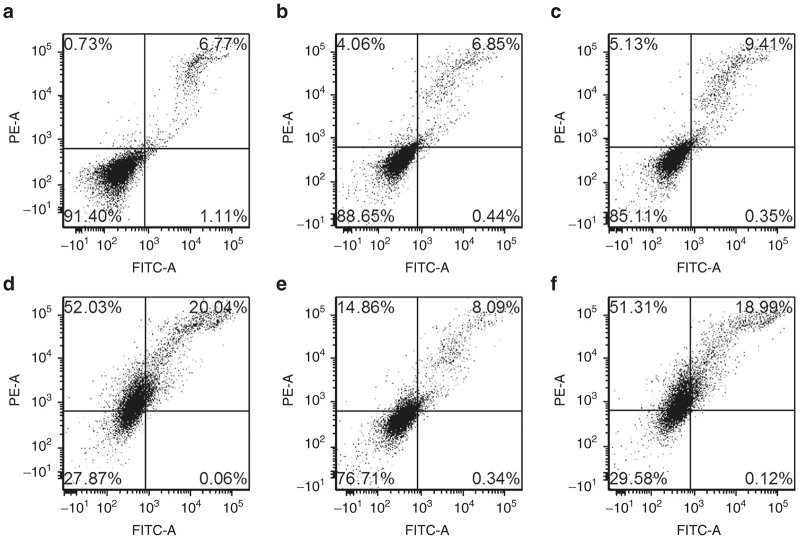
Flow cytometric analysis of cell death mode in CT26 cells by annexin V-FITC/propidium iodide (PI) double staining. Cells were either kept untreated (**a**) or treated with liposome of BIO-C18 containing RFC11 (**b**), Shikonin (**c**), and both RFC11 and shikonin (**d**) for 16 hours. Liposomes of BIO-C18 containing both RFC11 and shikonin exhibited synergistic cytotoxic effect which was attenuated by pretreatment with necroptosis inhibitor NEC-1 (**e**) but not upon pretreatment with caspase inhibitor Z-VAD-FMK (**f**).

Toward evaluating an insight into the mechanism of such synergistic inhibition of cell growth in CT26 colon carcinoma cells exhibited by liposome of BIO**-**C18 containing RFC11 and shikonin, flow cytometric analysis with Annexin V and PI double staining was performed. CT26 cells incubated with biotinylated liposomes containing both RFC11 and shikonin significantly enhanced the populations of necrotic and late apoptotic cells compared to cells treated with liposomal formulations of only RFC11 or shikonin (Figure 4). As shown in Figure 4, the percentage of necrotic and apoptotic cells were 52.03 and 20.04%, respectively. Interestingly, population of doubly stained cells in different quadrants remain unaltered upon pretreatment with caspase inhibitor Z-VAD-FMK (an inhibitor of apoptosis), indicating that the cell death is not due to induction of apoptosis and is caspase independent. However, pretreatment with Nec-1 (small molecule inhibitor of necroptosis) reduced the population of necrotic and late apoptotic cells to 14.86 and 8.09% suggesting the cell death mode to be necroptosis instead of apoptosis or necrosis. Such cytotoxic effect of RFC11 and shikonin coencapsulated in the liposome of BIO-C18 was not observed in noncancer cell (CHO) (Supplementary Figure S10). Taken together, these findings evidently suggest that N-end rule inhibitor RFC11 synergizes the cytotoxic effect of shikonin selectively in cancer cells via induction of necroptosis.

### Transmission electron micrographs of CT26 colon cancer cells

**Figure 5:**
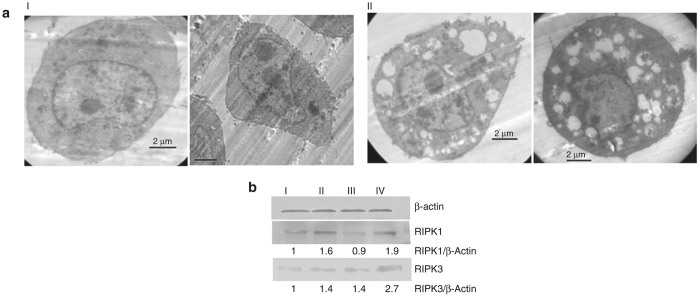
(**a**) Transmission electron micrographs of untreated CT26 cells (**I**) and cells treated with biotinylated liposomes co-encapsulated with RFC11 and shikonin (**II**). Morphological features of necroptosis (similar to necrosis) such as electron-lucent cytoplasm, swollen cytoplasmic organelles, intact nuclear membrane and initiation of loss of plasma membrane were found in CT26 cells treated with liposomes containing both RFC11 and shikonin (**b**). Western blot analysis of CT26 cell lysates: cell lysates were obtained from cells either kept untreated (**I**) or treated with liposomes of BIO-C18 lipid containing RFC11 (**II**), shikonin (**III**) or both RFC11 and shikonin (**IV**).

Morphological investigation by transmission electron microscopy was done to examine if the cells treated with liposomes of BIO-C18 containing both RFC11 and shikonin had characteristic features of necroptosis. As shown in Figure 5a, electron microscopic examination of untreated CT26cells revealed a smoothly outlined nucleus as well as well preserved plasma membrane and cytoplasmic organelles. By contrast, CT26 cells treated with liposomes of BIOC18 lipid containing both RFC11 and shikonin displayed typical necrotic cell death morphology (Figure 5a). As observed under transmission electron microscope, these cells displayed translucent cytoplasm, extensive vacuolation of cytoplasmic organelles, initiation of loss of plasma membrane integrity with an intact nuclear membrane. All these are typical morphological traits of necroptosis. No apoptotic morphological features like nuclear fragmentation and condensed cytoplasm was observed. These findings further confirmed the notion that synergistic inhibition of cell growth by liposomally bound RFC11 and shikonin is by induction of necroptosis and not apoptosis.

### Western blot analysis

To study the molecular mechanism behind synergistic induction of necroptosis in cancer cell by liposomally bound RFC11 and shikonin, we performed western blot analysis. It is already known that receptor-interacting protein 1 (RIP1) kinase is required to mediate necroptosis signaling through the activation of RIP3, a downstream mediator of necroptosis.^6^ Indeed, we found that expression level of RIP-1 and RIP-3 kinases was significantly upregulated in CT26 cells treated with liposomes of BIO-C18 containing RFC11 and shikonin (Figure 5b). Incidentally, RIPK1 expression was also increased by liposomally bound RFC11 but the corresponding cytotoxic effect was not observed in viability studies. However liposomal formulation containing both RFC11 and shikonin was more potent in upregulating the expression of RIP1 and RIP 3 kinases than liposomal formulation containing either RFC11 or shikonin.

### Tumor regression study

**Figure 6:**
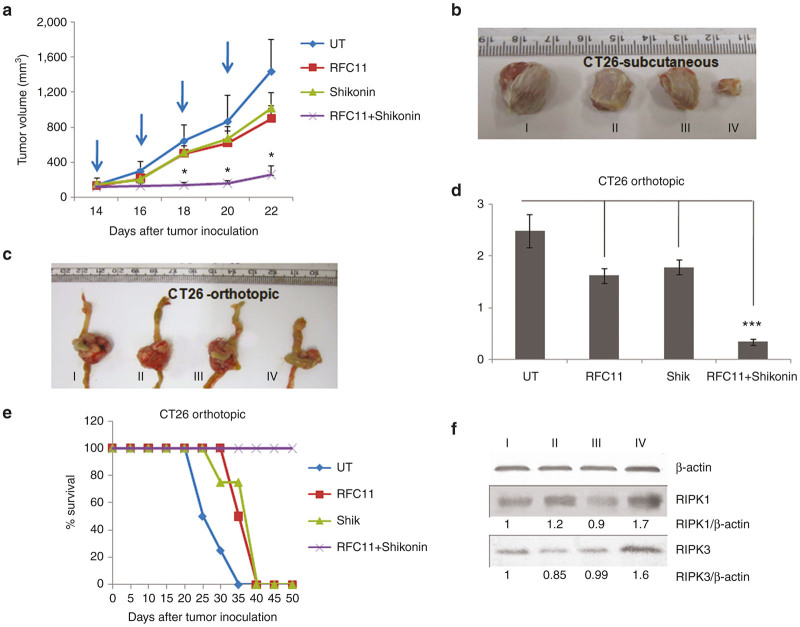
Biotinylated liposomes of RFC11 and shikonin inhibit tumor growth in mice. (**a**) Tumor regression curve after subcutaneous implantation of CT26 cells in BALB/c mice followed by intraperitoneal injection of 5% glucose, liposomes of BIO-C18 containing RFC11, shikonin (shik), both RFC11 and shikonin (*P < 0.01 compared with mice treated with 5%glucose only). (**b**) Representative subcutaneous tumors of CT26 cells in BALB/c mice excised on day 23. (**c**) Representative tumor pictures of orthotopically placed CT-26 tumors on day 23. Herein, (I), (II), (III), (IV) in both model denotes tumor from groups treated with 5% glucose, liposomes containing RFC11, liposomes containing shikonin, liposomes containing both RFC11 and shikonin, respectively. (**d**) Comparison of respective tumor weights obtained from various treatment groups in CT-26 orthotopic tumor model. The weight of each tumor is normalized by subtracting weight of a cecum obtained from fresh, nontumor-associated mice (***P < 0.003). (**e**) Survival graph of mice bearing orthotopic CT26 colon tumors and treated with different treatment groups. Intraperitoneal injection of treatments started from day 14 for both subcutaneous and orthotopic model and mice were administered five injections on every alternate day. (**f**) Western blot analysis of tumor lysates: Differential expression of different regulators of necroptosis in CT26 tumor lysates from the subcutaneous tumors of untreated group (I), from groups treated with BIO-C18 associated liposome containing RFC11 (II), shikonin (III) or both RFC11 and shikonin (IV).

Next, we assessed the observed *in vitro* synergistic effect of shikonin and RFC11 in *in vivo* condition. By using a previously reported subcutaneous and orthotopic colon carcinoma model of CT26 murine colon adenocarcinoma cells in BALB/c mice, we evaluated the tumor growth inhibiting efficacy of various liposomal formulation. Liposomes of Bio-C18 lipid containing both RFC11 and shikonin induced maximum tumor regression in both subcutaneous and orthotopic colon tumor models. The tumor volumes and weights of respective treatment groups in both the models clearly show the effective tumor-regression ability of shikonin and RFC11 encapsulated Bio-C18 liposome (Figure 6a–d). No significant tumor growth inhibition was found in mice treated with biotinylated liposomes carrying only RFC11 or shikonin respectively. Thus, the synergistic effects observed in tumor regression study are fully consistent with the *in vitro* findings. Also, coadministration of RFC11 and shikonin in biotinylated liposome enhanced the survival rate of *in situ* colon tumor bearing mice (Figure 6e). Contrastingly, tumor growth inhibition was found to be many folds less in mice treated with control nontargeting liposomal (void of BIO-C18) formulations containing RFC11 and shikonin individually or in combination (Supplementary Figure S11), indicating biotin receptors’ potential role in efficient delivery of drug cargoes.

### *In vivo* upregulation of RIP1 and RIP3 kinases

To study the effect of our liposomal formulation of RFC11 and shikonin in inducing necroptosis in tumor cells, we analyzed the expression levels of RIP1 and RIP3 kinases in tumor lysate. The higher level of RIP1 and RIP3 kinase in tumor lysate (Figure 6f) from group treated with biotinylated liposome containing RFC11 and shikonin suggest induction of necroptosis to be the plausible reason behind the observed tumor regression.

### Biodistribution studies

**Figure 7:**
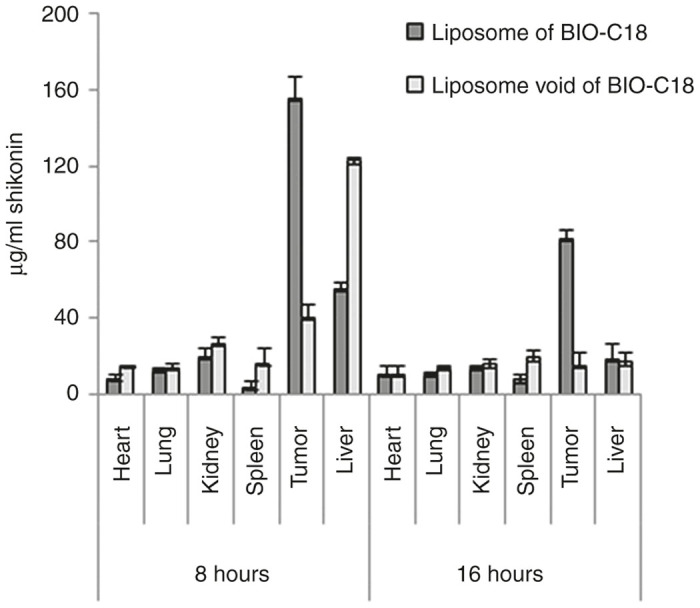
Functional biodistribution of liposomes of BIO-C18 lipid as well as its nontargeting counterpart (void of BIO-C18). Liposomes containing shikonin were intraperitoneally injected in mice bearing subcutaneous tumors of CT26 cells. Eight and 16 hours postinjection, mice were sacrificed and tumors and different vital organs were processed. Concentration of shikonin in each organ extract was quantified by measuring fluorescence at λ_emission_ 600 nm.

Toward evaluating the target specificity of our formulation, liposome of Bio-C18 lipid containing shikonin was injected intraperitoneally at a dose of 6 mg/kg B.W in BALB/c mice bearing subcutaneous tumor (~1,000 mm^3^) of CT26-injected cells. Fluorescence intensities in different organs were measured after 8 and 16 hours respectively (Figure 7). Figure 7 clearly shows accumulation of shikonin is strikingly high in tumor than in other vital organs. By contrast, shikonin entrapped in nontargeted liposomes (void of BIO-C18) was found to be majorly accumulated in liver but lesser in tumor mass. The drug biodistribution pattern fits in line with the tumor regression data (Figure 6; Supplementary Figure S11). The plasma concentration profile (Supplementary Figure S12) revealed that concentration of shikonin reached maxima at 60 minutes, and then decreased rapidly followed by a constant plasma concentration in the time range 2–8 hours. Thus, our designed liposomal formulation of Bio-C18 lipid is circulation stable at least for 8 hours and can be used to specifically deliver cytotoxic drugs to tumors.

## Discussion

The N-end rule pathway is an ubiquitin-dependent protein degradation pathway. It primarily relates the recognition of N-terminal amino acid residues (N-degrons) by specific E3 ubiquitin ligases.^15^ These E3 ligases bear two recognition domains (N-recognins), one of which recognizes proteins by their hydrophilic N-end amino acid (called Type 1 substrate with Arg, Lys, His as N-end) and other which recognizes proteins by their hydrophobic N-end amino acid (called Type 2 with Phe, Leu, Ile, Trp, Tyr N-end).^15^ N-degrons, recognized by N-recognins then undergo ubiquitination and 26S-mediated selective proteolysis. This pathway controls *in vivo* half-life of many proteins involved in many crucial physiological functions such as, regulation of cell cycle, import of peptides, chromosomal stability, cardiovascular development, apoptosis, etc.^30–32^ Piatkov *et al*. have recently shown that N-end rule pathway destroys many proapoptotic fragments toward restoration of cellular viability by counteracting death signals. Fragmented proapoptotic proteins like BRCA1 (N-Asp), RIPK1 (N-Cys), TRAF1 (N-Cys), BCL_XL_ (N-Asp), BID (N-Arg) contain destabilizing N-terminal residues and are shown to be degraded via N-end rule pathway.^14^

As stated above, like many other protein fragments, fragmented RIPK1, bearing Cys at its N-terminal, has been shown as N-end rule substrate and it degrades via *Arg-ATE1* N-end rule pathway.^14^ This RIPK1 (receptor interacting serine-threonine protein kinase-1) is one of the key regulators of necroptotic cell death.^33^ Necroptosis or programmed necrosis is recently emerging as an alternate therapeutic modality for apoptosis-resistant cancer cells and its initiation requires the activation of RIP1 and RIP3 kinases. Several necroptotic inducers like staurosporine, E6 analogs, shikonin are being studied to this end.^34,35^ However, to our knowledge, a potential synergistic chemotherapeutic approach that will take the advantage of N-end rule pathway inhibition toward inducing elevated necroptosis is never before exploited. Inhibition of N-end rule pathway by using small molecule inhibitors or some other means can stabilize necroptosis-inducing protein fragments, thereby sensitizing cancer cells to “programmed cell death” (apoptosis/necroptosis). Recently, RFC11, a lipid based hetero-bivalent inhibitor of N-end rule pathway was developed in our laboratory.^18^ Therein, we showed that RFC11 while inhibiting N-end rule pathway stabilized the N-end rule substrate and antiangiogenic factor RGS4 reduced cardiomyocyte proliferation and rendered these cells less hypertrophic. In RFC11, two C10 hydrocarbon chains were conjugated to two ligand heads; type 1 (Arg) and type 2 (Phe) amino acid residues, while hydrocarbon chains are attached to core lysine. The idea was to develop a stable inhibitor. As RFC11 is a lipid-based molecule, it is resistant to proteolytic degradation by cellular endopeptidase, thus acts as a stable inhibitor unlike dipeptide inhibitors (Arg-Ala, Phe-Ala) for N-end rule pathway.^36,37^ As discussed, RFC11 was able to stabilize the N-end rule substrate RGS4, which was one of the cardiovascular G protein signaling regulators involved critically in vascular maturation/integrity.^38^ Moreover, N-end rule pathway is independently proved to be involved in embryonic cardiac development and angiogenesis.^39^ So, hypothetically, N-end rule inhibitors may act in one hand as regulator of cardiovascular development and in other hand may have distinctive role in regulating angiogenesis. Angiogenesis is one of the hallmarks of cancer progression.^40^ On testing RFC11 on angiogenic model of HUVEC cells, we indeed find its ability to reduce cellular alignment of these cells, thus indicating negative effect of this molecule on HUVEC-based angiogenic model (data not shown). However, whether RFC11 had any direct effect on tumor was not evident then.

Fragments of RIPK1 (necroptotic or apoptotic factor depending on external stimuli), BRCA-1, TRAFF, BID, BCl_xl_ (proapoptotic factors) or RAD21 (important regulator of anaphase promoting complex), HDM2, IAP, Caspases, etc. are distinctive N-end rule substrates.^14,17,30^ Their physiological presence (or absence) is evolutionarily monitored by N-end rule pathway. Hence, physiological stabilization of proapoptotic N-end rule substrates would be a natural choice to understand the role of N-end rule pathway (if any) in cancer progression. If any inhibitor of N-end rule pathway can physiologically stabilize such proapoptotic factors, one can reduce the apoptotic threshold of cancer cells thus sensitizing them to externally treated drugs. Recently, we find that RFC11 can selectively manipulate/regulate the presence of XIAP and RAD21 by upregulating p53 in cancer cells, thereby making them drug sensitive (data not shown). However, elucidating the effectiveness of RFC11 in actual tumor model was a challenge. In this study, we used shikonin as codrug for RFC11 in two tumor models. Shikonin is a naturally occurring napthoquinone derived from Chinese medicinal herb *Lithospermum erythrorhixon* and is known to induce apoptosis or necroptosis depending upon cell type and concentration used. Huang *et al*. and Fu *et al*. have individually shown that shikonin induces RIP1- and RIP3-dependent necroptosis in glioma and osteosarcoma cells.^11,12^

At par with the hypothesis that inhibition of N-end rule pathway (by RFC11) can sensitize cancer cells to external drugs like shikonin, here in this study, we cotreated CT26 mouse colon adenocarcinoma cells with RFC11 and shikonin, both solubilized in a stable biotin receptor-targeted liposomal formulation. Such liposome has been formulated by using a synthesized amphiphile containing biotin in its polar head group in combination with other colipids. Biotin is an essential micronutrient required for normal cellular functions and its receptor is overexpressed in a number of cancer cell lines like ovarian, colorectal, lung, etc.^22–24^ The encapsulation of RFC11 and shikonin in the liposomal formulation overcomes various limitations associated with pristine delivery of hydrophobic drugs. Treatment of pristine drugs are inefficient and collaterally damaging because of drug’s poor bioavailability, nonspecific tissue distribution, etc. Although RFC11 forms micelle in aqueous medium^41^ but its media integrity and drug-encapsulation property are not established. Use of pristine drugs in “RFC11 only” formulation would also lack targeting ability. Toward this, we designed the biotin receptor-targeted liposomal system to simultaneously deliver shikonin and RFC11 to tumors overexpressing this receptor. The idea was also to make the formulation, which was preclinically tested in orthotopic and subcutaneous colon tumor model, clinically relevant. Biotin receptor helped in internalization of biotinylated liposome carrying combination drug thereby inducing targeted and synergistic cytotoxicity in cancer cells (as represented in isobologram plot in Supplementary Figure S9). By flow cytometry analyses, we found that biotin liposome-mediated delivery of combination drug led to more necroptosis in CT26 cells than the necroptosis induced by control liposome (Supplementary Figure S13). However, upon presaturation of cells with biotin, biotin liposome–mediated necroptosis was only partially inhibited and exhibited level of necrosis, which was equivalent to that of necrosis shown by control liposome (Supplementary Figure S13). This indicates that biotin receptor may have a role in surplus induction of necroptosis, which warrants further investigation. Upregulation of RIPK1 suggests possibility of both apoptosis as well as necroptosis but simultaneous upregulation of RIPK3 indicates prominent induction of necroptosis.^6^ Our data indicate that the combination drug treatment led to necroptosis, as the combination drug treatment triggered upregulation of both RIP1 and RIP3 kinases in CT26 cells. Clearly, the drug-sensitizing effect of RFC11 in cancer cells is noticeable which is reflected in its synergistic anticancer effect and for its very prominent simultaneous upregulation of RIPK1 and RIPK3 expressions, as are evident in cellular and tumor levels. In overall, in the age where intense efforts are made to overcome drug-resistance in cancer cells, selective delivery of N-end rule pathway inhibitor in cancer cells using a biotin receptor-targeted liposome that increases therapeutic potency of codelivered anticancer drugs can be an efficient mode of treatment in the field of cancer chemotherapy. Finally, our data opens up a basic question: how cancer modulates evolutionarily conserved N-end rule pathway-based proteasomal degradation machinery for its own sustenance and proliferation. The molecule RFC11 and the targeted delivery system as described here can act as probing system to understand the molecular pathogenesis of cancer *vis-á-vis* N-end rule pathway.

### Conclusion

In conclusion, in this study, we have inhibited N-end rule pathway to sensitize cancer cells against anticancer drugs. The concept was verified in animal tumor model. We have developed a biotin receptor-targeted circulation stable liposomal formulation for codelivering N-end rule inhibitor RFC11 and anticancer drug shikonin to cancer cells via biotin receptor. This liposomal system can find future use for simultaneous delivery of hydrophobic drugs or small molecules to cancer cells. Also, we expect that N-end rule pathway inhibitors (such as RFC11) can play a bigger role in developing novel therapeutics against cancer.

## Materials and Methods

### Reagents and antibodies

Shikonin, 3-(4,5-Dimethylthiazol-2-yl)-2,5-diphenyltetrazolium bromide (MTT), Propidium Iodide (PI), FITC (fluorescein isothiocyanate)-labeled Annexin V, cholesterol, BCIP/NBT (5-bromo-4-chloro-3-indolyl-phosphate/nitro blue tetrazolium) substrate, and RIPA buffer were purchased from Sigma, St. Louis, MO. HBTU and DSPE-PEG (2000)-NH_2_ were procured from Novabiochem and Avanti Polar Lipids, respectively. Unless otherwise stated, all chemicals, reagents, and organic solvent were purchased from local commercial suppliers and were used without further purification. Anti- receptor interacting protein (anti-RIP) antibody (ab72139) and Anti-RIP3 antibody (ab152130) were purchased from Abcam. β-actin antibody (13E5) was procured from Cell Signaling Technology. Goat anti-mouse IgG Alkaline Phosphatase (DC05L) and goat anti-rabbit IgG Alkaline Phosphatase (DC06L) were purchased from Calbiochem.

### Syntheses

RFC11 was synthesized using the same procedure as described before.^18^ Details of the synthetic steps for preparing biotin lipid derivative (BIO-C18) are shown in Supplementary Figure S1. The starting amine namely, N-2-aminoethyl-N,N-di-n-octadecylamine was prepared as described previously.^42^ Structures of all the synthetic intermediates shown in Supplementary Figure S1 were confirmed by ^1^H NMR and ESI-MS. The purity of the BIO-C18 lipid was confirmed by reversed phase analytical high performance liquid chromatography (HPLC) analysis.

Step (i): Succinic anhydride (452.3 mg, 4.52 mmol) was suspended in dioxane and N-2-aminoethyl-N,N-di-n-octadecylamine (849 mg, 1.5 mmol) was slowly added to it keeping the reaction mixture in ice bath for 5 minutes. The resulting reaction mixture was stirred at room temperature for further 16 hours. Dioxane was then evaporated in the rotary evaporator at 55 °C and the mixture was purified by column chromatography using 60–120 mesh silica gel and 5–7% methanol-chloroform (v/v) as eluent. This yielded intermediate (**1**) as pure white solid. (802 mg, 80% yield, R_f_ = 0.2 in 5% methanol-chloroform (v/v). ESI-MS: 665.6 [M+1]^ +^ for C_42_H_84_N_2_O_3._

Step (ii): D-Biotin was coupled with N-BOC-1,2-Diaminoethane according to the procedure previously reported.^43^ Briefly, 4 mmol D-biotin, 5 mmol N-BOC-1,2- diaminoethane and 5.4 mmol 1-Ethyl-3-(3-dimethylaminopropyl)carbodiimide (EDC) in 10 ml MeOH and 30 ml CH_3_CN was stirred for 12 hours at room temperature. The mixture was then concentrated, re-suspended in methanol and filtered over celite. The filtrate was concentrated and the purified by column chromatography to give the desired product in 50% yield.

Step (iii) and (iv): To a solution of N-BOC-N-(2-(D-Biotinylamino)-ethyl) – amine (compound **2**, 500 mg, 1.29 mmol) in 5 ml dichloromethane (DCM), 5 ml trifluoroacetic acid (TFA) was added drop-wise at 0 °C. The resulting mixture was stirred at 0 °C for 3 hours to ensure complete BOC-deprotection. Excess TFA was removed by nitrogen flushing. This afforded the free amine in high purity (as revealed by TLC i.e., thin layer chromatography) and it was directly used in next step. ESI-MS: m/z = 287.29 [M+1]^+^. The resulting amine (350 mg, 1.3 mmol, 95% yield) was dissolved in 5 ml dimethylformamide (DMF) and the solution was added to an ice-cold turbid reaction mixture containing solid HBTU (2-(1H-benzotriazol-1-yl)-1,1,3,3-tetramethyluronium hexafluorophosphate, a coupling reagent) (221 mg, 0.98 mmol) and compound **1** (651 mg, 0.98 mmol) in 5 ml DCM. Diisopropylethyl amine (DIPEA) was added drop-wise to the stirred reaction mixture kept in ice until it became alkaline to litmus. The resulting solution was then stirred at room temperature for 16 hours. The reaction mixture was then diluted with 100 ml chloroform and sequentially washed with ice cold water (3 × 100 ml), ice-cooled 0.5 N HCl (2 × 100 ml), saturated sodium bicarbonate (2 × 100 ml) and brine (1 × 100 ml). The organic layer was collected, dried over anhydrous sodium sulfate, filtered, and concentrated by rotary evaporation. The residue was initially purified by column chromatography using 60–120 mesh silica gel and 3% methanol-chloroform (v/v) as eluent. Further purification by recrystalization with methanol/diethyl ether afforded final desired compound BIO-C18lipid (500 mg, 43.8% yield) in high purity. The final BIO-C18 lipid was characterized by ESI-MS, ^1^H NMR and its purity was confirmed by reversed phase analytical HPLC using methanol as mobile phase (Supplementary Figures S2–S4). ^1^H NMR (300 MHz, CDCl_3_ + CD_3_OD): δ/ppm = 0.9 (t, 6H), 1.3–1.4 (m, 66H), 1.6 (m, 4H), 2.18–2.96 (m, 12H), 3.2–3.3 (m, 9H), 4.3–4.5 (m, 2H), 7.3 (s, 5H). ESI-MS: 933.8 [M+1]^+^ for C_54_H_104_N_6_O_4_S

### Preparation of liposomes

Liposomes were prepared by lipid thin film hydration technique. Briefly, (n-C_16_H_33_)_2_N^+^(CH_3_) CH_2_CH_2_N^+^(CH_3_)_3_2Cl^−^(cationic lipid obtained as a gift from the laboratory of Dr. Arabinda Chaudhuri, CSIR-IICT, Hyderabad, India and later synthesized indigenously), cholesterol, biotin amphiphile (BIO-C18) and DSPE-PEG-NH_2_ in the mole ratio of 1:0.5:0.05:0.01 were dissolved in chloroform and methanol (5:1 v/v) in a glass vial. 25 mole% (with respect to cationic lipid) of RFC11 and 4 mole% (with respect to cationic lipid) of shikonin was added in the liposomes containing RFC11, shikonin or both RFC11 and shikonin. The solvent was removed by a thin flow of nitrogen and the dried lipid film was kept under high vacuum for 4 hours. The mixture was hydrated with 1 ml of sterile water overnight. It was then vortexed for 2 minutes, bath sonicated at low intensity for 5 minutes and finally probe sonicated for 2 minutes in ice using a constant duty cycle and output control magnitude of 2–3 in a Branson sonifier 450. Liposomes were centrifuged at 5,000 rpm for 30 minutes to remove unencapsulated drug. Concentration of entrapped shikonin was then quantified spectrophotometrically (Biotek SYNERGY H1 micro plate reader) at 520 nm (characteristic absorbance of shikonin) after lysing the liposomal solutions with 1% Triton-X inmethanol. The entrapment efficiency was then calculated as the drug loaded in the liposomes divided by total drug used. Drug-loading efficiency of the liposomes was found to be in the range of 90–95% routinely. 1 mmol/l liposome (with respect to cationic lipid) was used for all in vitro studies. For animal studies, 5 mmol/l liposome (with respect to cationic lipid) dispersed in 5% glucose solution was used.

### Characterization of liposomes

The particle size and zeta potential of liposomes diluted in deionized water were determined by dynamic light scattering analysis using a Zetasizer Nano-ZS (Malvern Instruments, Malvern, UK) at room temperature. Also, the morphology of liposomes was examined under a high-resolution transmission electron microscope (JEOL-JEM 2100). Briefly, 5 μl of sample was placed on carbon-coated copper grid (glow discharged for 45 seconds using Tolaron Hivac Evaporator). After 10 minutes, excess sample was blotted away by Whatman filter paper. Next, 5 μl of 2% uranyl acetate was added, kept for 2 minutes and the grid was air dried before analyzing at 120 KV.

### Serum stability of liposomes

To examine the serum stability of liposomes of BIO-C18 lipid, various liposomal formulations were incubated in PBS containing 20% and 50% fetal bovine serum (v/v) at 37 °C. Dynamic light scattering was used to measure the size of liposome mixture at definite time points ranging from 0 to 72 hours.

### Cell line and cell culture

CT26 (mice colon adenocarcinoma syngeneic to BALB/c) was procured from ATCC. A549 (Human lung adenocarcinoma), Hela (Human cervical cancer), CHO (Chinese hamster ovary), NIH-3T3 (mouse embryonic fibroblast) cells were purchased from National Center for Cell Sciences (Pune, India). HFF (human foreskin fibroblast) cell was a kind gift from CCMB, Hyderabad. All the cells were checked to be mycoplasma free. CT26 cells were grown in Roswell park memorial institute (RPMI) medium (PAN Biotech, Aidenbach, Germany) while A549, Hela, HFF, CHO, and NIH-3T3 cells were expanded in Dulbecco’s modified Eagle’s medium (Sigma) both supplemented with 10% fetal bovine serum (FBS) (South American Origin, Lonza) and 1% penicillin–streptomycin–kanamycin at 37 °C in a humidified atmosphere of 5% CO_2_ in air.

### Cellular uptake study

Cellular uptake of liposomes of BIO-C18 lipid as well as control liposomes (without Bio-C18) was studied by labeling the liposomes with Rho-PE (0.1 mole% with respect to lipid). A549, Hela, HFF and CT26 cells were seeded at a density of ~10,000 cells per well in a 96-well plate before treatment with Rho-PE labeled liposomes. Two hours post-treatment, the cells were washed with PBS (3 × 100 µl) and live cells were finally imaged under an inverted fluorescence microscope (Nikon, Japan).

### Biotin receptor selectivity study

CT26, A549, Hela, and HFF cells were seeded at a density of 1.0 × 10^4^ cells per well in a 96-well plate 18–24 hours before treatment. Cells were preincubated with 1 mmol/l Biotin in Dulbecco’s modified Eagle’s medium containing 10% FBS for 1 hour and then coincubated with Rh-PE-labeled liposomes. After 2 hours of incubation, cells were washed with PBS (3 × 100 µl) and the live cells were imaged under an inverted fluorescence microscope. Uptake of Rh-PE-labeled liposome in CT26 cells was further quantified by flow cytometry analysis. Briefly cells were seeded in six-well plate at a density of ~2 × 10^5^ cells per well, 18 hours prior to treatment. Cells were similarly preincubated with 1 mmol/l Biotin for 1 hour followed by coincubation with Rh-PE lipososme for 2 hours. Cells were then trypsinized, washed with PBS, and the cellular uptake of Rh-PE was analyzed by flow cytometry.

### *In vitro* cell cytotoxicity studies

Cellular cytotoxicities of biotinylated liposomes containing RFC11 and/or shikonin were evaluated by the standard 3-(4,5-dimethylthiazol-2-yl)-2,5-diphenyltetrazolium bromide (MTT) reduction assay. Briefly, cells were seeded at a density of 5,000 cells/well in a 96-well plate 18–24 hours before experiment. Treatments were done to triplicate wells. Cells were treated with liposomes containing RFC11, liposomes containing shikonin and liposomes containing both RFC11 and shikonin continuously for 24 hours. Following the termination of experiment, cells were washed and promptly assayed for viability using MTT. Results were expressed as percent viability = (A_550_(treated cells)–background/A_550_(untreated cells)–background) × 100

### UBR1 downregulation by siRNA delivery

CT26 cells were seeded at a density of 2 × 10^5^ cells per well in a six-well plate 18–24 hours before transfection. 10 pmol of UBR1-siRNA (Dharmacon) or 10 pmol of scrambled siRNA (Dharmacon) (diluted to 50 μl with serum-free RPMI) was complexed with 2 μl of Lipofectamine2000TM (Invitrogen) (diluted to 50 μl with serum-free RPMI) for 15 minutes. 900 μl serum-free RPMI was added to the resulting complex and added to cells. After 4 hours, serum-free media were discarded and serum-containing RPMI was added. After 36 hours, cells were trypsinized and again seeded at a density of 5,000 cells per well in 96-well plate for cytotoxicity assay and rest of the cells were seeded in 60 mm Petri-dish. After 12 hours, 96-well plate cells were either kept untreated or treated with liposomes of BIO-C18 containing RFC11 (5 µmol/l) shikonin (1 µmol/l) or combination of RFC11 and shikonin (5 and 1 µmol/l, respectively). Cytotoxicity assay was performed using MTT as described earlier. In 60 mm Petri-plate, cells were either kept untreated or treated with RF-C11 (5 µmol/l) for 12 hours. The cells were then lysed to get whole cell lysate for western blot analysis.

### Drug synergism study

Isobologram analysis was done to study drug synergism. The isobologram was plotted as a graph of equally effective drug dose pairs (isoboles) into a single effect level. Selectively, a particular inhibitory concentrations of drug B and drug A (each alone) are chosen (here, 90% of the maximum (IC90)), resulting doses of combination effect are plotted as axial points in a Cartesian plot. The antagonistic, additive, and synergistic effects can be evaluated by calculating combination index (CI-value) using mathematical formula from isobologram. Formula and significance of CI-value is given below. Herein, ICx,A and ICx,B are the inhibitory concentration × (ICx) of drug A and drug B. The points of ICx of A and B were added by a straight line. The points at which concentrations of drug A and B meet is called as combination index (CI), which was calculated using this equation.

CI = CA, X/ICx, A + CB, X/ICx,B

Here, CI < 1 indicates synergism effect, CI = 1 indicates additive effect, CI > 1 indicates antagonism effect

### Flowcytometric analysis for assessing mode of cell death

The annexin V-FITC-labeled apoptosis detection kit (Sigma) was used to detect the cell death mode (apoptosis or necroptosis) by flow cytometry as per manufacturer’s protocol. In brief, CT26 and CHO cells (1.5 × 10^5^ cells/well) were seeded in six-well plates and cultured over night in RPMI and Dulbecco’s modified Eagle’s medium medium, respectively with 10% fetal bovine serum. After 16–18 hours, cells were either kept untreated or treated with liposomes of BIO-C18 lipid containing RFC11, shikonin, or both RFC11 and shikonin (5 and 1 µmol/l) for 16 hours. For CT26 cells, two other groups were treated with 10 µmol/l Nec-1 (inhibitor of necroptosis) and 20 µmol/l z-VAD-fmk (caspase inhibitor) for 4 hours prior to coincubation with liposome containing both RFC11 and shikonin. Then, the cells were trypsinized, collected by centrifugation for 5 minutes at 2,000 rpm and resuspended in 1× binding buffer containing FITC-labeled Annexin V (25 ng/ml) and propidium iodide (50 ng/ml). Cells were analyzed using a flow cytometer (FACS Canto II) and data were analyzed with FACS Diva software. A minimum of 10,000 events was gated per sample.

### Transmission electron microscopy

CT26 cells were cultured and expanded in RPMI medium supplemented with 10% fetal bovine serum in tissue culture flasks (75 mm^2^). When the cells reached 60–70% confluence, cells were either kept untreated or treated with liposomes of BIOC18 coencapsulated with RFC11 and shikonin. Sixteen hours post-treatment, cells were trypsinized, washed with PBS, and fixed using 2.5% glutaraldehyde. After 24 hours, cells were again washed with PBS followed by postfixation with 1% OsO4. The samples were next dehydrated using varied percentage of acetone, embedded in epoxy resin and allowed for polymerization at 60 °C. Thin sections were cut on 15 Ultramicrotome (Leica, Germany), placed on mesh of copper grids, and stained with 2% Uranyl acetate, followed by counter staining with lead citrate. Micrographs were visualized on transmission electron microscope (JEOL-JEM 2100), operating at 120 KV.

### Western blot

For this study, CT26 cells were seeded in tissue culture flask (25 mm^2^) and expanded in RPMI medium with 10% fetal bovine serum until they reached 60–70% confluence. Cells were either kept untreated or continuously treated for 12 hours with liposomes of BIO-C18 lipid containing RFC11, shikonin, and both RFC11 and shikonin. Following treatment, cells were detached from flask using a cell scrapper and lysed using RIPA buffer at 4 °C. For preparation of tumor lysate, one mouse with representative tumor size from each group was sacrificed after completion of *in vivo* experiment in BALB/c mice. Whole cell lysates from four different treatment groups were prepared as follows: a part of tumor were suspended in 500 μl PBS, homogenized using a mechanical homogenizer and centrifuged at 10,000 rpm for 10 minutes. The supernatant was discarded; 250 μl RIPA buffer premixed with Protease inhibitor cocktail antipain and leupeptin (Calbiochem) was added to the cell pellet and kept in ice for 30 minutes with occasional tapping. Total protein contents of the cell lysates were quantified by BCA protein estimation kit (thermo pierce) and 60 µg of total proteins were dissolved in SDS-PAGE sample buffer prior to separation by 12% SDS-PAGE. The protein bands were transferred to PVDF membrane and immunoblotted. Antibody-reactive bands were detected by BCIP/NBT substrate (Sigma Aldrich) as alkaline phosphates conjugated secondary antibodies (Goat-anti-rabbit and Goat-anti-mouse) were used.

### Tumor growth inhibition study

Five- to six-week-old male BALB/c mice (~20g) were obtained from NIN (Hyderabad, India) and kept in an animal house maintaining institutional animal safety guidelines. ~1.5 × 10^5^ CT26 cells in 250 μl PBS were injected subcutaneously in the right flank of mice on day 0. For orthotopic tumor model, 1 × 10^5^ CT26 cells in 100 μl PBS were surgically injected into the cecal wall of BALB/c mice. Fourteen days following tumor cell implantation in both models, mice were randomly sorted in groups and each group (*n* = 5) was administered intraperitonially with liposomes containing (i) RFC11 (8 mg/kg B.W), (ii) shikonin (0.75 mg/kg B.W), and (iii) both RFC11 (8 mg/kg B.W) and shikonin (0.75 mg/kg B.W). The control group (*n* = 5) was treated with 5% glucose only. Each mouse received five intraperitoneal injections on every alternate day. The tumor volumes were measured with slide calipers and calculated using the formula 0.5 × a × b^2^, where a is the longest dimension and b is the shortest dimension of the tumors. Experiment was terminated when the average tumor volume of the untreated group reached 3000 mm^3^. The protocols for animal experimentations were approved through Institutional Animal Ethical Committee. The photographs of the representative tumors in both subcutaneous and orthotopic models were taken on day 23.

### Functional bio-distribution and pharmacokinetics

Male BALB/c mice (*n* = 3) were subcutaneously injected with ~1.5 × 10^5^ CT26 cells in 250 μl PBS in right flank. When tumor volume reached ~1,000 mm^3^, mice were intraperitonially injected with a single dose of biotin receptor targeted liposomal shikonin and nontargeted liposomal shikonin (6 mg/kg body weight). Eight and 16 hours postinjection, mice were sacrificed and all vital organs including tumor were collected. 500 μl lysis buffer (0.1 M Tris–HCl, 2 mmol/l EDTA and 0.2% Triton X-100, pH 7.8) was added to each organ and homogenized using a mechanical homogenizer. The homogenates were extracted twice with 1 ml ethyl acetate by vortex mixing for 5 minutes at high speed and centrifuged at 10,000 rpm for 10 minutes. The upper organic layer was collected and evaporated to dryness. The residues were dissolved in 200 μl methanol and fluorescence of 100 μl of the extract was measured at λ_emission_ 600 nm (Biotek SYNERGY H1 microplate reader). To correct for tissue auto fluorescence, untreated control tissues were similarly extracted. For each sample, the background fluorescence was subtracted and the fluorescence intensities in different organs were converted to μg/ml of shikonin using a known standard curve of shikonin. For pharmacokinetic study, blood samples were collected in K_2_EDTA-coated tubes via retro orbital puncture at definite time intervals (0–8 hours) after i.p. administration of the above liposomes in tumor bearing BALB/c mice (*n* = 3). Plasma was obtained from the blood samples by centrifugation at 3,000 rpm for 5 minutes. Hundred microliters of plasma were then mixed with 10% triton-X and the mixture was extracted with ethyl acetate by vigorous vortexing for 5 minutes and then centrifuged at 5,000 rpm. Blank plasma from untreated mice was similarly extracted. The organic layer was collected, evaporated to dryness and the residue was dissolved in 100 μl methanol. The amount of shikonin in blood plasma was measured using a spectrofluorimeter.

## Figures and Tables

**Figure 8:**
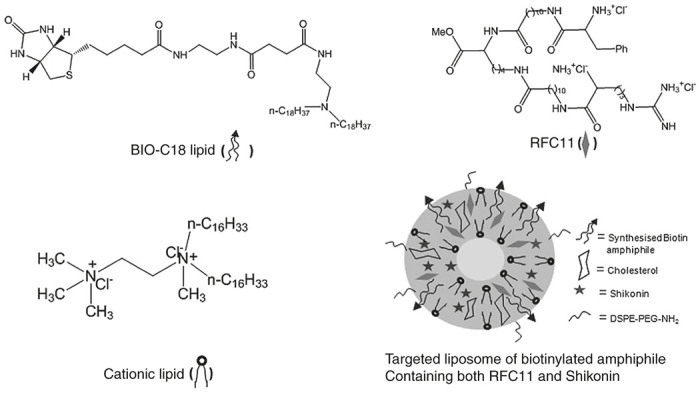
Schematic representation of individual structures of targeting lipid BIO-C18, N-end rule inhibitor RFC11, constituent cationic lipid and the theme structure of targeted liposomal formulation.
