# Supplementary material for: N-end rule pathway inhibition assists colon tumor regression via necroptosis
Source: Mol Ther Oncolytics. 2016 Aug 10;3:16020–. doi: 10.1038/mto.2016.20 (PMC4980110; doi:10.1038/mto.2016.20)
Supplement: Supplementary Information [file mto201620-s1.docx]

SUPPLEMENTARY INFORMATION

**N-end rule pathway inhibition assists colon tumor regression via necroptosis**

*Pritha Agarwalla, Rajkumar Banerjee*

Reagents: (i) succinic anhydride, dioxane, 16 h; (ii) N-BOC-1,2-Diaminoethane, EDCI, methanol-acetonitrile (1:3 v/v), 12 h ; (iii) 50 % TFA in DCM, 3 h, 0°C; (iv) HBTU, DMF-DCM (1:1, v/v), intermediate **1**, DIPEA.

**Figure S1.**Scheme used for synthesis of **BIO-C18** lipid


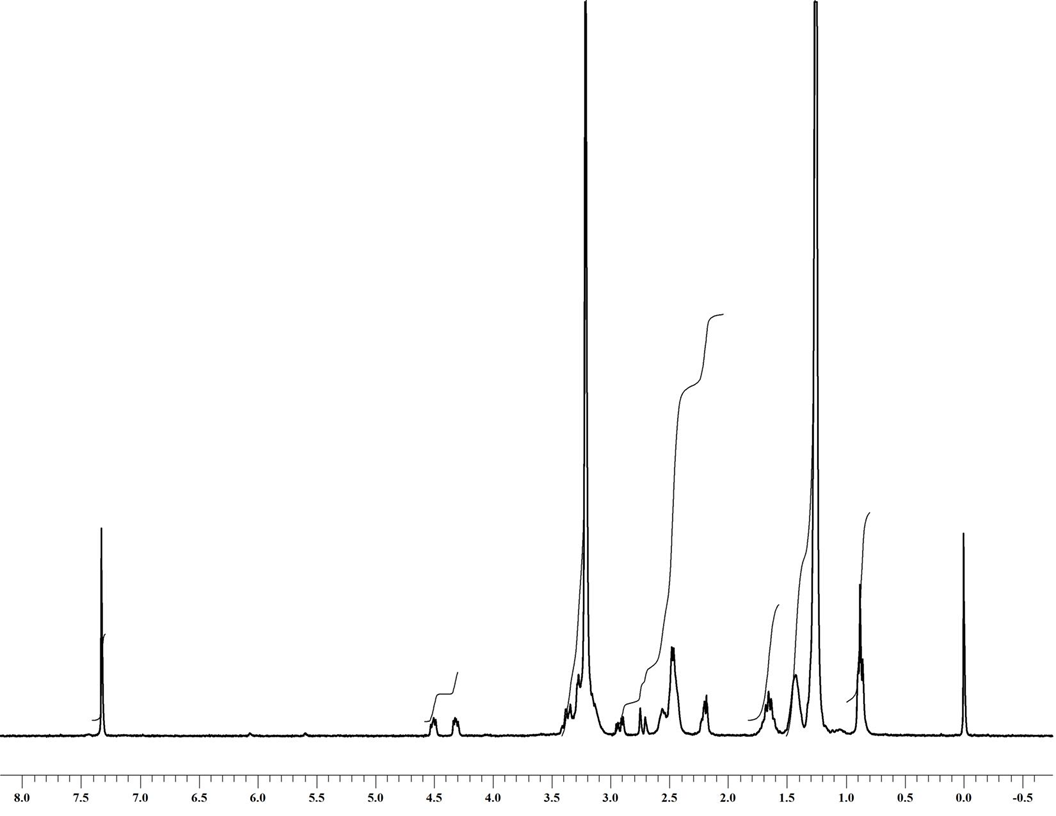
**Figure S2.**^1^H NMR (300 MHz) of BIO-C18 lipid

**
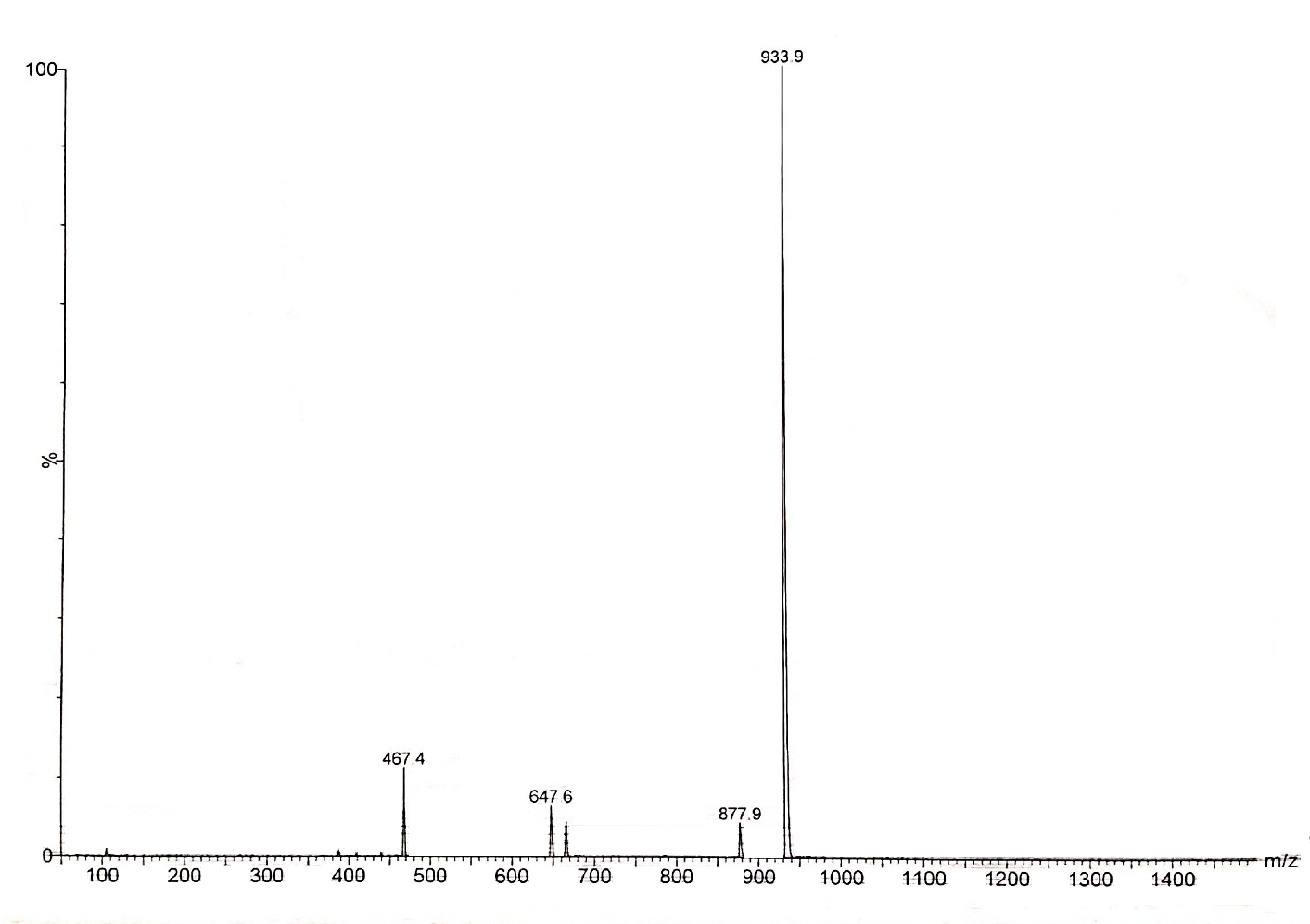
**

**Figure S3**. ESI-MS of BIO-C18 lipid

**
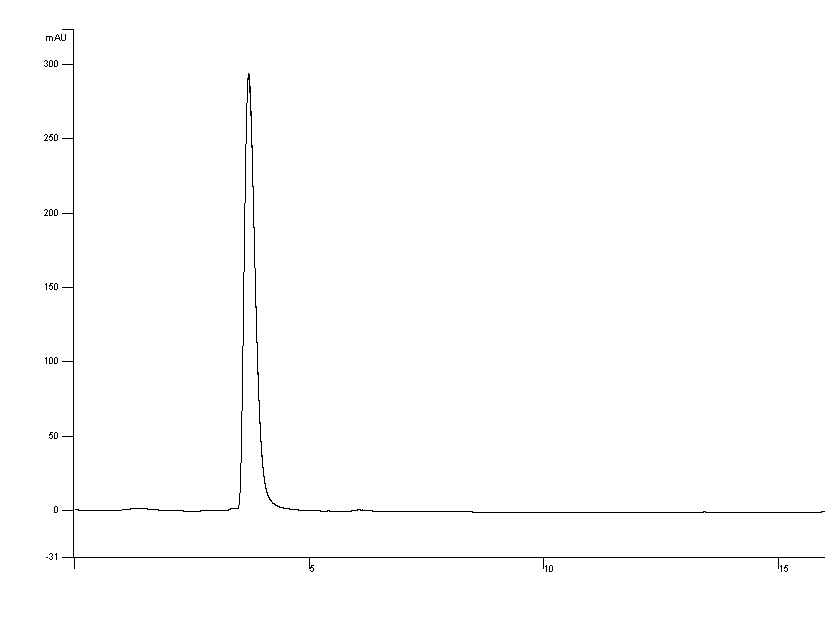
**

**Figure S4.**HPLC Chromatogram of BIO-C18 lipid**.**

**HPLC Conditions:**System: Varian 1100 series, Column: Lichrospher® 100, RP-18e (5 µm), Mobile Phase: Methanol,Flow Rate: 1.0 mL/min (0-15 min)**,** Typical Column Pressure: 60-65 Bars, Detection: UV at 210 nm

**Supplementary table 1**: Sizes and Zeta potentials of liposomes of BIO-C18 containing RFC11 and/or shikonin.

| liposomes | Hydrodynamic diameter (nm) | Zeta potential (mv) |
| --- | --- | --- |
| Biotinylated liposomes containing RFC11  Biotinylated liposomes containing shikonin  Biotinylated liposomes containing both RFC11 and shikonin | 155±3  164±4  218±2 | 27±4  22±2  31±4 |


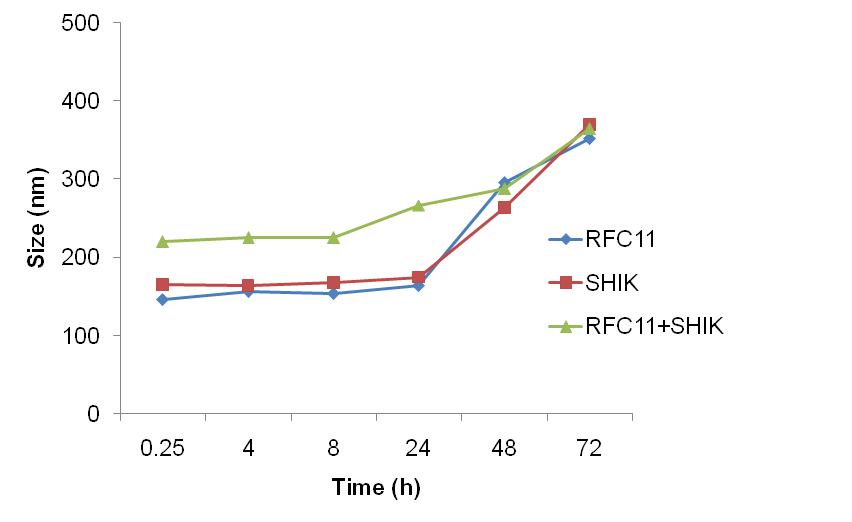

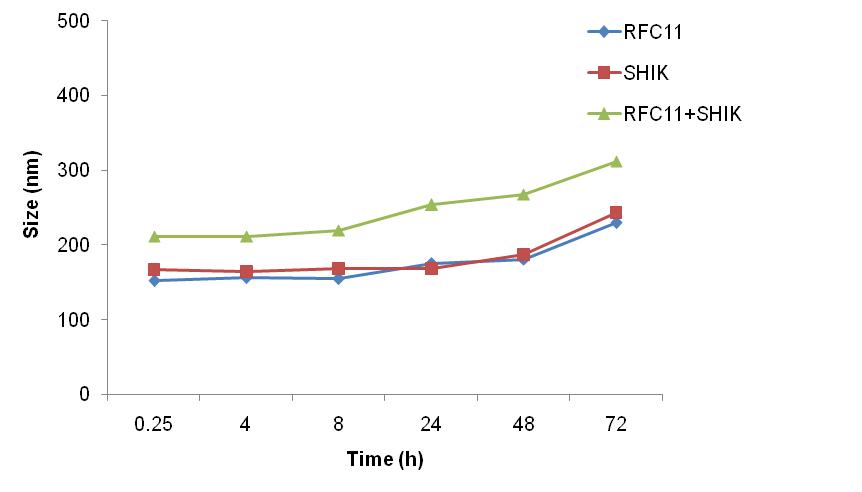


**B.**

**A.**

**Figure S5**. Serum stability: Changes in size of various liposomes of BIO-C18 lipid upon incubation in 20% (A) and 50% (B) serum at 37 ºC.

**Figure S6**. Flow cytometric uptake analysis of Rh-PE liposome (red) which decreased considerably when cells were pre-saturated with biotin when compared to untreated (black)

**Figure S7**. Microscopic images showing cellular uptake of Rh-PE labeled liposomes of BIO-C18 lipid (panel A) which decreased considerably upon pretreatment with 1 mM biotin (panel B) in biotin receptor over expressed in A549 cells (**I**) and Hela cells (**II**) but not in normal HFF cells (**III)**. All the images were taken 2h after treatment with liposomes at 10X magnification.


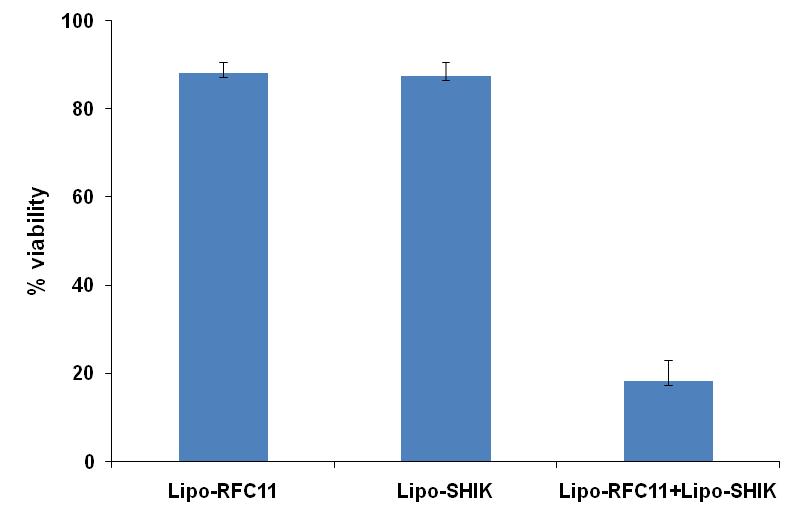


**Figure S8.** Cell viability studies: Co-treatment of RFC11 and shikonin individually encapsulated in liposomes of Bio-C18 exhibited synergistic cytotoxic effect in CT26 cells.

**Figure S9.** Isobologram analysis showing that RFC11 and shikonin cotreatment in biotinylated liposome synergistically induced cell death in CT26 cells.

**A.**

**B.**

**D.**

**C.**

**Figure S10.** Flow cytometric analysis of cellular cytotoxicityin CHO cells co-stained with annexin V-FITC/ propidium iodide (PI). Cells were either kept untreated (A) or treated with biotinylated liposomes containing 5μM of RFC11 (B), 1 μM Shikonin (C) and (5+1)μM of (RF-C11+Shikonin) (D) for 16h.

**Figure S11. (A)** Tumor growth curve after subcutaneous implantation of CT26 cells in BALBc mice followed by intaperitonial injection of 5 % glucose or non-targeting control liposomes entrapped with RFC11 or shikonin individually or in combination**.** Intraperitonial injection started from day 14 and five injections were given every alternate day. The asterisk (*) denotes p <0.01 while comparing with UT. (**B**) Representative tumor pictures in each group after sacrificing mice on day 23. Herein, (I), (II), (III), (IV), denotes tumors from groups treated with 5 % glucose, liposomes containing RFC11, liposomes containing shikonin, liposoems containing both RFC11 & shikonin respectively.

**Figure S12.** Time-course of shikonin concentration in plasma determined at 0.25, 0.5, 1, 2, 4 and 8 h post injection of liposomes of BIO-C18 lipid.

**A.**

**D.**

**E.**

**B.**

**C.**

**Figure S13**. Assessment of cell death mode by flow cytometric analysis using annexin V-FITC/ propidium iodide (PI) double staining. Cells were either kept untreated (A) or treated with liposome of BIO-C18 (B) or control liposomes void of BIO-C18 (C) both containing RFC11 & Shikonin. Effect of biotin pre-incubation on cytotoxic effects exhibited by BIO-C18 liposomal formulation (D) or control liposome formulation (E). The extent of necroptosis by biotin receptor targeted formulation upon pre-treatement of biotin led to only partial ablation of necroptosis and exhibited necroptosis level equivalent to that triggered by control liposome with or without the pre-treatment of biotin. This indicates that this vitamin receptor may have a possible role in surging necroptosis in cancer cells.

.
